# Immune escape mutations in HIV-1 controllers in the Brazilian Amazon region

**DOI:** 10.1186/s12879-020-05268-0

**Published:** 2020-07-25

**Authors:** Samara Tatielle Monteiro Gomes, Ednelza da Silva Graça Amoras, Érica Ribeiro Gomes, Maria Alice Freitas Queiroz, Edivaldo Costa Sousa Júnior, Janaína Mota de Vasconcelos Massafra, Poliana da Silva Lemos, João Lídio Vianez Júnior, Ricardo Ishak, Antonio Carlos Rosário Vallinoto

**Affiliations:** 1 Laboratory of Virology, Biological Science Institute, Federal University of Pará (ICB/UFPA), Ananindeua, Brazil; 2 Graduate Program in Biology of Infectious and Parasitic Agents, Biological Science Institute, Federal University of Pará, Ananindeua, Brazil; 3 Health Surveillance Department, Ministry of Health (IEC-SVS/MS), Evandro Chagas Institute, Ananindeua, Brazil

**Keywords:** HIV-1, Viremia controllers, Escape mutations

## Abstract

**Background:**

Human immunodeficiency virus (HIV-1) infection is characterized by high viral replication and a decrease in CD4^+^ T cells (CD4^+^TC), resulting in AIDS, which can lead to death. In elite controllers and viremia controllers, viral replication is naturally controlled, with maintenance of CD4^+^TC levels without the use of antiretroviral therapy (ART).

**Methods:**

The aim of the present study was to describe virological and immunological risk factors among HIV-1-infected individuals according to characteristics of progression to AIDS. The sample included 30 treatment-naive patients classified into three groups based on infection duration (> 6 years), CD4^+^TC count and viral load: (i) 2 elite controllers (ECs), (ii) 7 viremia controllers (VCs) and (iii) 21 nonviremia controllers (NVCs). Nested PCR was employed to amplify the virus genome, which was later sequenced using the Ion PGM platform for subtyping and analysis of immune escape mutations.

**Results:**

Viral samples were classified as HIV-1 subtypes B and F. Greater selection pressure on mutations was observed in the group of viremia controllers, with a higher frequency of immunological escape mutations in the genes investigated, including two new mutations in *gag.* The viral sequences of viremia controllers and nonviremia controllers did not differ significantly regarding the presence of immune escape mutations.

**Conclusion:**

The results suggest that progression to AIDS is not dependent on a single variable but rather on a set of characteristics and pressures exerted by virus biology and interactions with immunogenetic host factors.

## Backgrounds

The majority of patients infected with human immunodeficiency virus (HIV) present high viral replication rates and a reduction in the number of CD4^+^ T cells (CD4^+^TC), which in the absence of antiretroviral therapy may lead to the development of acquired immune deficiency syndrome (AIDS) and ultimately death [1]. However, in some infected individuals, viral replication is apparently controlled without the use of antiretroviral drugs.

Long-term nonprogressor (LTNP) cases comprise an HIV-1 infection group that does not follow the normal course of AIDS progression due to the ability to spontaneously maintain high levels of CD4^+^ T cells and a low viral load in the absence of therapy [2]. These individuals can be subclassified into groups of viremia controllers (VCs) and elite controllers (ECs), which represent the extreme capacity to curb progression to AIDS [3].

However, the nomenclature and classification criteria of these groups vary according to the author [2]. Currently, the suppression mechanism of viral infection remains poorly understood and is commonly attributed to virological factors, in addition to other factors such as immune response, genetic variations and gut translocation, which are directly associated with the inflammation process [4, 5].

Variants of human leukocyte antigen (HLA) genes can influence the effectiveness of the host immune response [6]. For example, the presence of protective HLA alleles, such as HLA-B*57 and HLA-B*27, is associated with lower viral loads and slower progression to AIDS, whereas the HLA-B*35 allele is associated with rapid progression to the disease [7–11].

Viremia controllers usually display a trend toward an immune response consisting of a Th1 profile, with a significant increase in cytokine production and a reduction in the Th2 response compared with individuals who exhibit the usual progression to AIDS [12–14]. Although high production of IFN-γ is associated with slower progression to AIDS, IL-4 production is reportedly higher in individuals with rapid progression to AIDS [11], indicating that the Th1/Th2 balance is essential for determining the course of viral infection [13].

Julg [15] reported data indicating that CD4^+^ T cells from ECs are easily infected with HIV ex vivo, which suggests that these cells are not resistant to infection and that the virus isolated from these individuals exhibits competent replication. Another study isolating viral particles from ECs observed that the viruses of these individuals show normal replication kinetics in vitro, showing that defective viral strains are not the main cause of this phenomenon [16].

The virological characteristic most commonly associated with disease progression is a high level of HIV-1 mutation, which results from the reverse transcription process and contributes to a continuous increase in genetic diversity of the virus, allowing it to adapt quickly to a variety of selection pressures imposed by the hostile and changing environment of the host [17–19]. Indeed, host immune factors, including the response of cytotoxic CD8^+^ T lymphocytes (CTLs), exert a major selective force that drives the evolution and diversification of HIV-1 at both individual and population levels [7, 20, 21].

Thus, the CTL response plays a central role in the control of HIV infection [22]. Several studies have demonstrated that mutations allowing escape from the CTL response generally lead to rapid progression to AIDS [23–25]. In contrast, viral escape triggered during initial infection results in subsequent control of viremia [26–29]. In general, the emergence of escape mutations during the immune response provides a clear physical benefit to the virus but is associated with costs to viral fitness [30–32].

The present study attempted to describe immune escape mutations related to structural (*gag*), regulatory (*tat* and *rev*) and accessory (*nef*, *vif*, *vpr* and *vpu*) genes in treatment-naive HIV-1-positive individuals with different profiles of progression to AIDS and to correlate the mutations with these progression outcomes.

## Methods

### Population characteristics

The present study employed a cross-sectional retrospective design and used 30 samples from patients with confirmed HIV-1 infection belonging to the collection of the Virus Laboratory of the Biological Science Institute of the Federal University of Pará. The samples were collected in a referral unit in the city of Belém, Pará state, Brazil, and sent to the laboratory through the Brazilian National Network for CD4^+^/CD8^+^ T Cell Count and Viral Load of the Ministry of Health. The samples were collected from 2007 to 2011 using a vacuum collection system with 5-mL tubes containing EDTA as an anticoagulant and stored at − 20 °C in the Laboratory of Virology of UFPA until tests were performed.

The subjects had no history of antiretroviral therapy prior to the time of sample collection and were distributed into three groups, elite controllers (ECs), viremia controllers (VCs) and nonviremia controllers (NVCs), according to previously described criteria for progression to AIDS [12]. These criteria consisted of HIV-1 infection for six or more years, CD4^+^ T cell counts above 500 cells/mm^3^ for controllers and below this level for NVCs and viral loads below 50 copies/mL (not detectable) for ECs, below or equal to log_4_ for VCs and greater than log_4_ for NVCs. Based on these criteria, 9 individuals were classified as controllers (ECs and VCs) and 21 as nonviremia controllers (NVCs).

### Molecular analysis

DNA extraction, genome amplification and purification of products.

Total DNA was extracted from whole blood using the Biopur Mini Spin Plus extraction kit (Biometrix, Brazil). The HIV-1 genome was amplified by nested PCR using previously described primers [33]. The amplification reaction was performed in a volume of 50 μL containing 10 ng of extracted DNA, 200 mM of each dNTP, 200 nmol of each primer, 50 mM KCl, 1.5 mM MgCl_2_, 10 mM Tris-HCl (pH 8.3) and 1 U of Taq DNA polymerase for both rounds. The amplification products were visualized after electrophoresis (100 V/45 min) through a 1.5% agarose gel. To remove impurities and nonspecific products that might interfere with sequencing, the products were purified from agarose gel slices using PureLink Quick Gel Extraction Kit (Thermo Fisher Scientific, USA). Commercial assays were used according to the technical procedures recommended by the manufacturer.

### Sequencing, editing and alignment

Sample sequencing was carried out using an Ion PGM™ System (Life Technologies, Carlsbad, California, USA) platform based on the Ion Xpress Plus gDNA Fragment Library Preparation protocol (Life Technologies, Carlsbad, California, USA), which is capable of processing long DNA sequences and generating high coverage rates. Samples were loaded onto an Ion 314 chip (Life Technologies, Carlsbad, California, USA) with an output per run of up to 100 Mb. Nucleotide editing and alignment were performed to obtain consensus sequences using Geneious 8.1.2 software [34]. The genome was manually curated using the HIV-1 viral genome (NC001802) as a reference for mapping. Only high-quality sequences with phred values greater than 20 were included in the analysis.

### Phylogenetic analysis and identification of escape mutations

The sequences generated were subjected to phylogenetic analysis for the determination of HIV-1 subtypes. Phylogenetic inferences were performed using the Bayesian MCMC (Markov Chain Monte Carlo) method and BEAST 1.8.2 software [35], with 100 million generations and resampling at each 10,000 generations. Convergence analysis was estimated with ESS over 200. For analysis of the molecular clock, the strict model was applied using the GTR (General Time Reversible) model of nucleotide substitution. The coalescence model used was expansion growth with triplicates of 100 million generations.

The presence of CTL escape mutations was analyzed using Geneious 8.1.2 software [33], comparing the investigated amino acid sequences with epitope data available from Los Alamos HIV Immunology Database (https://www.hiv.lanl.gov/content/index) and sequences previously described in the literature. For *gag*, 24 types of mutations were investigated, as were 6 in *rev*, 7 in *tat*, 11 in *nef*, 9 in *vif*, and 4 in *vpr* and *vpu*.

### Three-dimensional protein modeling

Three-dimensional protein structure was constructed using protein modeling by homology with Modeler 9.15 software [36]. An initial search and selection of templates was conducted using the Protein Data Bank (https://www.rcsb.org/pdb/home/home.do), applying the HIV-1 p24 protein sequence as the initial parameter. The template selected was 5HGK. Visualization and production of the images were carried out using PYMOL 1.8 software [37].

## Results

**Fig. 1:**
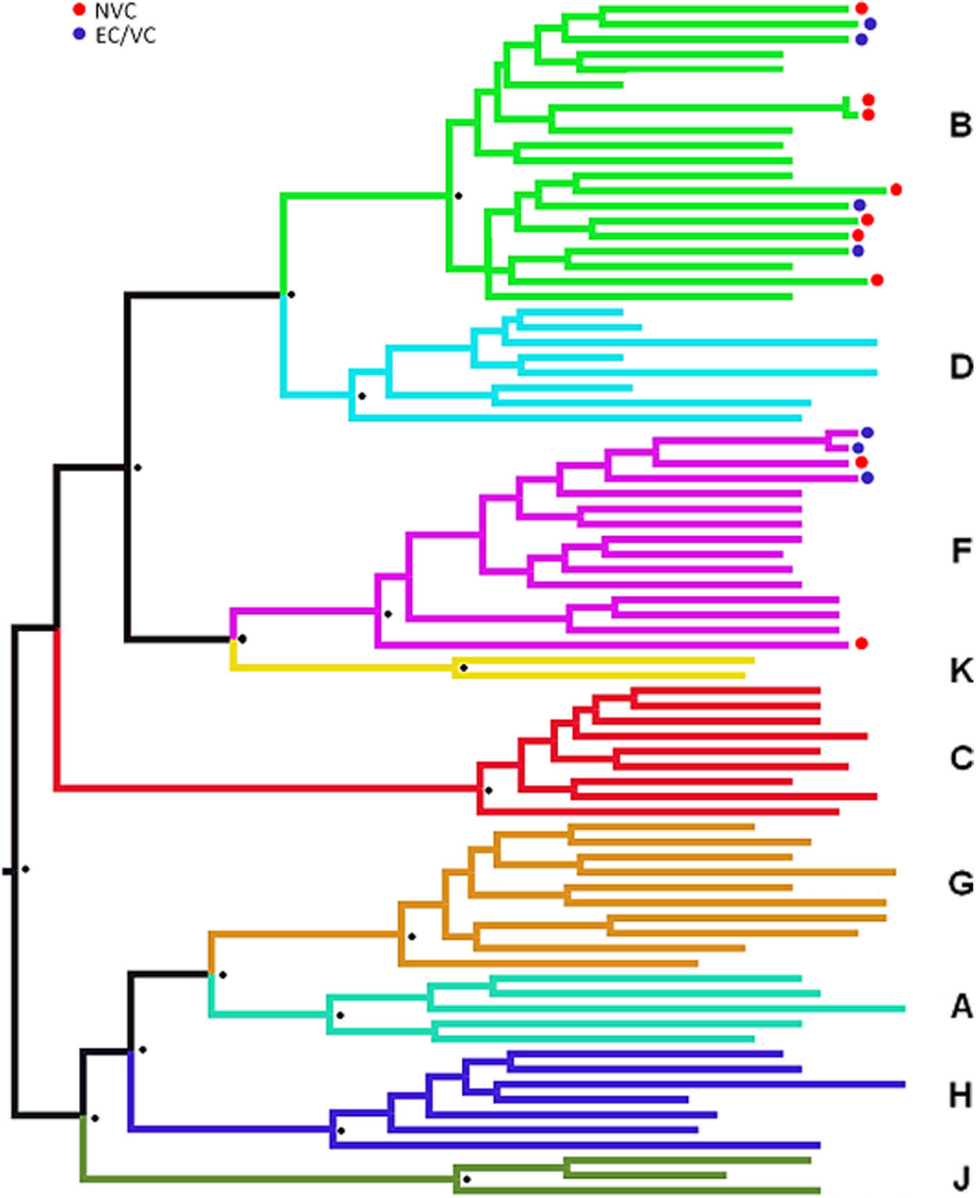
Phylogenetic distribution of the analyzed samples. Identification of individuals from the EC/VC (elite and viremia controllers) and NVC (nonviremia controllers) groups of different HIV-1 subtypes

**Fig. 2:**
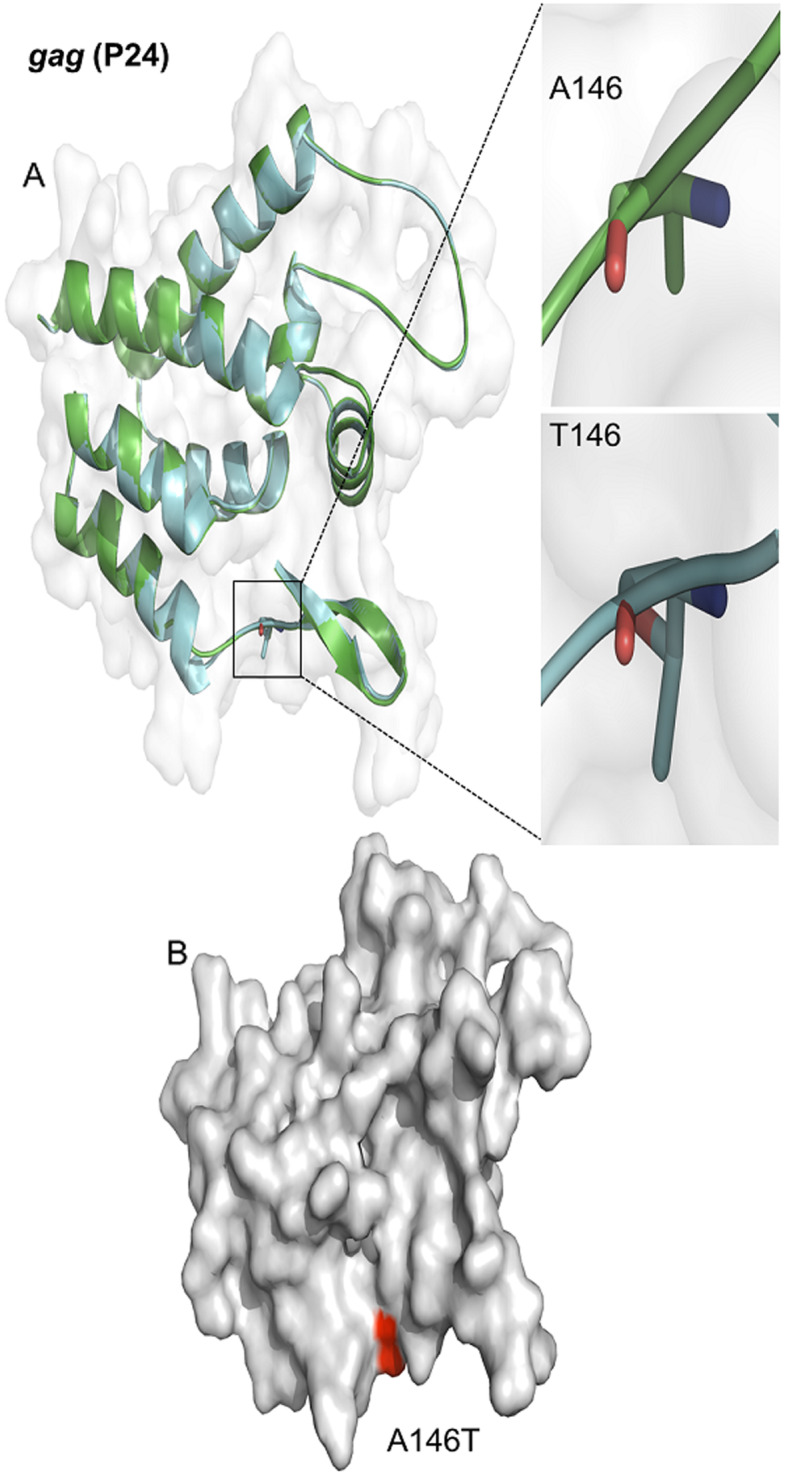
Location of the A146T mutation in the P24 protein encoded by the HIV-1 *gag* gene. A - Enlargement of the substitution site of alanine (A146) to threonine (T146) at position 146. B - Three-dimensional representation of the P24 protein, with the location of the A146T mutation indicated

Phylogenetic analysis of *gag* included 16 samples, for which good-quality sequences were obtained, grouped into subtypes B (68.75%) and F (31.25%). EC/VC groups (ECs and VCs; *n* = 7) were of subtypes B (57.14%; 4/7) and F (42.86%; 3/7). Nine samples for the NVC group were sequenced, with 77.78% (7/9) of subtype B and 22.22% (2/9) of subtype F (Fig. 1). There were 12 CTL escape mutations detected in each group (Table 1). In addition, two new mutations were identified, namely, a substitution from alanine to threonine at position 146 of the p24 protein (A146T; Fig. 2) in two individuals from the VC group and a phenylalanine substitution to leucine at position 383 of the p7 protein (F383L) in the NVC group.

**Table 1:**
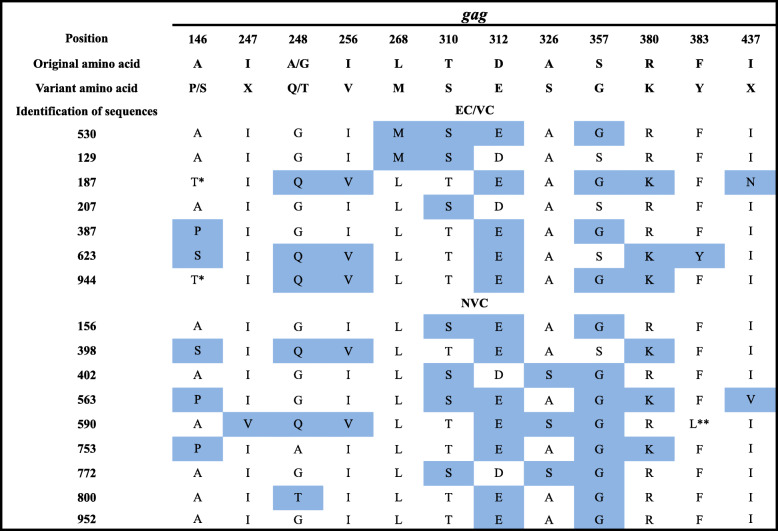
Immune escape mutations found in the HIV-1 *gag* gene. ECs/VCs (elite and viremia controllers); NVCs (nonviremia controllers)

Boxes in blue denote amino acid substitutions. * New mutation previously described in the literature. ** New mutation not previously described in the literature

*Rev* sequences were examined in 10 individuals: 2 ECs, 2 VCs and 6 NVCs. Two mutations were found at positions 17 and 21 in VCs, and one mutation was found at position 2 in six NVC individuals. Evaluation of *tat* included 9 sequences (from 1 EC, 3 VCs and 5 NVCs) and revealed the presence of 6 mutations at positions 7, 9, 24, 29, 32 and 36 in VC individuals and 3 mutations at positions 24, 29 and 36 in NVCs (Table 2).

**Table 2:**
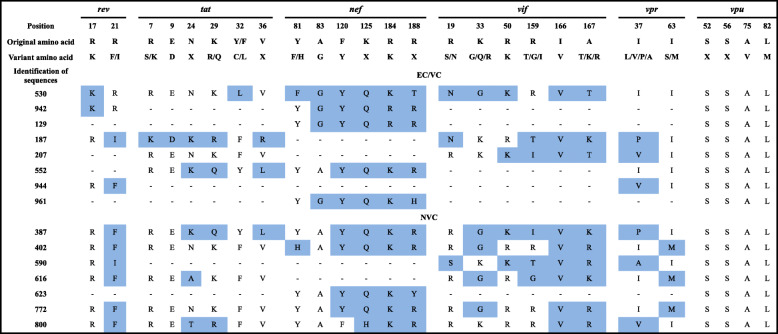
Immune escape mutations found in the regulatory and accessory genes of HIV-1. ECs/VCs (elite and viremia controllers); NVCs (nonviremia controllers)

Boxes in blue denote amino acid substitutions. Dashes indicate the lack of sequence for the gene

*nef* sequences from 10 individuals were assessed, including 5 VCs (2 ECs and 3 VCs) and 5 NVCs. One EC individual carried 6 escape mutations, and the others presented between 4 and 5 mutations. Five mutations were detected among NVCs. Nine samples (1 EC, 2 VCs and 6 NVCs) were analyzed with regard to *vif*, revealing 6 escape mutations among both groups. The highest number of sequences obtained was for *vpr,* with a total of 11 (1 EC, 4 VCs and 6 NVCs). Only one type of mutation in VCs and two types in NVCs were identified. Moreover, no resistance mutations in *vpu* were found among 15 samples (2 ECs, 7 VCs and 7 NVCs) (Table 2).

## Discussion

For most people with HIV-1 who do not receive ART, the infection progresses to AIDS in approximately 2 to 4 years, but other outcomes have been observed [2]. These different forms of progression are determined by complex interactions between genetic factors of the host and poorly understood intrinsic characteristics of the virus [4, 21, 38–40].

Studies of viremia controllers provide a unique opportunity to understand the mechanisms involved in the natural control of HIV-1 infection and to clarify factors that affect disease progression in infected individuals, providing crucial insight for the development of more effective therapeutic strategies. This is especially relevant for the study of mechanisms that may block viral infection and for the development of an effective vaccine [4, 16].

HIV-1-positive individuals in whom disease progression is controlled in the absence of ART, such as members of EC and VC groups, are rare, and the mechanisms involved in establishing this phenotype have not yet been fully elucidated. Nonetheless, despite undetectable plasma viral loads (< 50 copies/mL), ultrasensitive methods have shown baseline levels of replication in these patients, which allows some level of viral evolution due to the high potential of genetic variability in HIV-1 [41].

Phylogenetic analysis of sequences among viremia and nonviremia HIV-1 controllers was performed using the *gag* gene region. Subtypes B and F were distributed among 16 samples, with no common origin for slow progressor sequences, excluding a possible shared ancestry of reduced virulence [42]. In Brazil, subtype B is most prevalent, followed by subtype F [43, 44]. These results are partially in agreement with those reported by Machado [45], who analyzed *env* and *pol* gene regions and described the presence of subtypes C and D (in addition to subtypes B and F) in individuals visiting a reference unit in the states of Pará and Amapá. The absence of these subtypes in our study may be due to the small sample size.

A more robust Th1 immune response profile may lead to greater selection pressure on the viral population in such controllers [3, 46, 47]. This was previously observed with the same group of individuals examined in the present study [12], Thus, the selection pressure of the CTL response acts as a driving force throughout infection, resulting in the emergence of immune escape mutations, especially among those carrying HLA alleles not associated with clinical progression [48–50]. For all genes investigated, immune escape mutations were present at a higher frequency among viremia controller groups (ECs and VCs). The sole exception was *gag*, which showed a similar distribution in both the EC/VC and NVC groups. These mutations have been related to the presentation of antigens by MHC class I, which activates the CD8^+^ T lymphocyte response [51].

Mutations observed in *gag* were mainly in p24, a conserved protein. Thus, CTL escape mutations occurring in this region are under strong selection pressure [52]. A total of 24 mutations of greater relevance were investigated, and 12 were present in the groups (one EC, six VCs and nine NVCs). For example, a mutation at position 146 (A146X/P/S) is commonly related to viral escape from epitope processing [53, 54]. However, the change from alanine to threonine found in the present work has not been previously described in human samples. Genetic manipulation of the virus has indicated that different approaches for modifying *gag* (including the mutation in question) produce a virus with significantly lower replication capacity in cell culture [55].

Among the mutations detected in the TW10 epitope, the replacement of threonine for asparagine at residue 242 (T242N) represents a well-known and important CTL escape mutation associated with HLA-B*57B and results in loss of recognition of the epitope by CTLs; its presence suggests a virus with compromised replication capacity [56]. Furthermore, previous studies have identified the A/G248 mutation as being associated with T242N-induced escape compensation because it partially induces the recovery of replicative ability; regardless, the T242N mutation was not found in this work [56, 57].

The A/G248 mutation, when it occurs alone, does not represent a consistent variant of viral escape and results in partial loss of epitope recognition. Moreover, it is usually associated with subtype B infections and has little or no implication for virus replication, especially when the host has an HLA phenotype that does not exert a high selection pressure on the immune system. Similarly, isoleucine at position 256 alone does not represent loss of viral fitness; however, when both mutations are present in the same individual, the result is more effective viral escape, irrespective of the HLA type of the host [56].

The KK10 epitope of p24 is an immunodominant region related to HLA B*27, and the appearance of escape mutations within this epitope correlates with increased viral replication and progression to AIDS [24, 58]. Mutations in this epitope were identified in two individuals (1 EC and 1 VC) in this study, which is contrary to the progression profile in which they were classified. Nonetheless, it has previously been reported that the virus can be controlled in individuals with the HLA B*57 genotype even in the presence of this mutation [28], an indication that these two controllers may harbor a protective HLA, though this was not directly investigated in the present study.

The QW9 epitope decreases recognition by CD8^+^ T lymphocytes [59]; it is more commonly found among subtype B sequences, with a higher incidence in progressors than in long-term nonprogressors (LTNPs). Nevertheless, Migueles [20] reported no differences in cytotoxic responses between LTNPs and progressors. It is possible that variations in a specific response may be associated with synergy of the QW9 epitope with other mutant epitopes, as the effect of a single epitope may not be an accurate representation of the global effects on the patient’s CD8^+^ T lymphocyte response to HIV-1.

The RI9 epitope is closely related to HLA B*13; this epitope is located in the p1 protein encoded by *gag* and has no specific assigned function. However, the p1 sequence is part of the viral RNA ribosomal frameshifting site, which acts directly on cleavage of the precursor polyprotein Gag-Pol. It is thought that changes in this region affect assembly of the viral particle, generating an accumulation of unprocessed Gag-Pol precursors [60, 61]. A previous study showed that variations in residues 436 and 437 of this Gag epitope are responsible for reducing CTL recognition and significant loss of replicative capacity in mutants [62]. In the present study, variations at residue 437 were observed in an EC/VC and in an NVC, and it is not possible to attribute the advantage or disadvantage of this modification for progression to AIDS.

A change from phenylalanine to leucine at position 383 of p7 in *gag*, not previously described in the literature, was identified in an NVC subject in the present work. However, a substitution at the same position to tyrosine related to loss of recognition by the CTL response has been previously documented [51]. In addition to other critical functions for viral replication, the protein has a structural role in HIV-1 nucleocapsid formation. Modifications in this region may result in a defective, abnormally assembled mutant, which may lead to internalization or increased exposure of viral epitopes, thereby hindering recognition or increasing the reactivity of the protein. This would directly affect interactions with the immune system [63, 64].

Accessory and regulatory genes were also examined for CTL escape mutations. The *rev* gene is essential for viral replication, as it is responsible for the export of unprocessed or incompletely processed viral RNA from the nucleus to the cytoplasm. Two changes were found at positions 17 and 21 in a basic domain (originally rich in arginine). Its function involves specific binding to a region of the secondary complex of RNA, called the RRE, that mediates the export of mRNAs from the nucleus to the cytoplasm, where they are translated to produce essential viral proteins [65]. The greatest diversity of mutations at position 17 was present in the EC/VC group, which presented lower viral loads, corroborating the study of Rousseau [66], who reported an association between the presence of this variant and lower viral loads.

Six of the seven mutations investigated in *tat* were more frequent in the EC/VC group. The Tat protein is particularly effective at controlling viral replication in vivo [67–69]. The CC8 epitope is an inducer of immune escape from the CTL response. In a longitudinal study analyzing mutants at 15 different timepoints of sample collection from an HIV-positive individual in the first 3 years of infection, a 41% increase in the probability of survival and replication was observed compared to wildtype [70]. Mutation at position 32 (NY9) in the Tat protein sequence blocks processing of the MY9 epitope positioned at an adjacent region; this epitope shows high immunogenicity in subjects with the related HLA B*15 genotype. Rapid elimination from the viral pool of direct and complete selection of escape mutations in the NY9 and MY9 epitopes is attributed to CTL responses [71].

The Nef protein is involved in reduced expression of CD4 receptors on the surface of infected cells and, consequently, contributes to rapid progression to AIDS [72], and *nef* sequences of the NVC group were more conserved than were those in the EC/VC group. This higher frequency of mutations in nonprogressors was reported by Kirchhoff [73], who analyzed *nef* gene sequences from five LTNP patients and found a high number of mutant forms that resulted in attenuation of HIV-1 particles. Immune escape mutations in this region generate a virus with low fitness, contributing to the absence of disease progression [74].

The *vif* gene encodes a protein that is central to viral replication because of its ability to neutralize the host’s antiviral APOBEC3 protein [42]. Vif optimizes viral replication capacity and acts as an integral component of the reverse transcription complex by serving as a cofactor of the reverse transcriptase enzyme, which is relevant for determining the timeline of progression to AIDS [75]. Hassaïne [76] demonstrated that a tyrosine kinase (Hck) protein was able to inhibit the production and infectivity of a virus with mutant *vif* but not the wildtype virus, showing the role of certain mutations in the loss of protein efficacy. In an analysis of sequences from individuals from northern India, Ronsard [77] observed that mutations at positions 166 and 167 of *vif* affect viral infectivity, and Rousseau [66] associated a variant at position 33 with lower viral loads, suggesting a cost of mutation to viral fitness. Similarly, a case study in the United States investigated viral mapping of a mother and daughter (congenital transmission) who were slow progressors and identified a two-amino acid insertion in Vif that reduced the in vitro replication capacity of the virus in PBMCs by 20-fold*.* Moreover, after reversal of the mutation, the replication efficiency was restored to levels equivalent to that of the wildtype virus, suggesting a mechanism of loss of Vif function. Furthermore, mapping revealed high identity between the viruses of the mother and daughter, even though 15 years had passed since the child’s birth, evidencing the sustained maintenance of the mutant virus. This may have been because it caused a milder infection [78]. These mutations in *vif* were observed in both progression groups in the present work. In addition, all were present in EC/VC sample 530, which corresponds to an EC.

The Vpr protein is associated with a variety of biological functions, including cell maintenance in G2 phase, induction of apoptosis, nuclear import of the preintegration complex, modulation of gene expression and suppression of immune activation [79]. Four mutations in this protein described in the literature have been analyzed, with changes at positions 37 and 63. HIV-1-positive patients with Vpr mutations exhibit slower progression to AIDS [80–82] as a consequence of its effects on the deregulation of major immune pathways, including antigen presentation, cytokine production and T cell activation [83, 84]. Nonetheless, the direct effects of Vpr on infected T cells and their immune functions are not clearly understood.

Consistent with the present study, such mutations were observed by Hadi [85], who analyzed 192 sequences from LTNP individuals and 102 fast progressors available in the Los Alamos sequence bank. The authors identified a change from isoleucine to serine (position 63) only in fast progressors; at position 37, there was no difference in the frequency of mutation between the groups, similar to the distribution observed in our study for both mutations. These results indicate that mutations in the *vpr* gene may impair cell cycle blockade at the G2 stationary phase and are more common in LTNPs than in fast progressors. G2 blockade likely provides favorable conditions for viral gene expression, which facilitates viral replication in addition to inducing immune escape, thus avoiding detection by NK cells because it facilitates the formation of viral reservoirs. Regardless, the mechanisms involved in the above phenomena are not yet clear.

Analysis of mutations in the *vpu* gene for both progression groups demonstrated high conservation of sequences, and no amino acid changes were found. The Vpu protein is a potent antagonist of the CD317/tetherin restriction factor, which limits the release of viral particles, modulates expression of MHC I receptors on the cell surface, regulates degradation of newly synthesized CD4 molecules still in the cell cytoplasm and further decreases activation of NK cells [86, 87]. Chen [88] was not able to demonstrate significant differences between the sequences from individuals with different forms of progression. The authors suggested that the lack of differences between the two groups may have been due to the wide importance of the function exerted by the Vpu protein, whereby any mutation in the region would result in strong selection pressure on the mutant protein by the immune system, hindering establishment of the mutation.

Based on the results presented herein, it is possible to suggest that the progression of HIV-1-induced disease is regulated by several viral and host factors operating in synergy. The high incidence of escape mutations in HIV-1 controllers is at least partially related to the cumulative effect of these mutations on viral fitness. Several studies have associated CTL escape mutations in p24 Gag with slower disease progression as a result of virus fitness incurred by these mutations [21, 57, 89, 90].

This study demonstrates that CTL responses in hosts are likely to have a strong influence on viral evolution during HIV-1 infection. This scenario provides a preliminary indication that therapeutic vaccines that induce robust responses to conserved epitopes, such as p24, may increase selection pressure on the virus, thus decreasing viral replication over the long term.

Caetano [91] compared ECs without detectable viremia and VCs with low quantified viral load blades and showed a higher frequency of mutations in the *gag* region in the former and *nef* in the latter, which might indicate a differential pattern of viral load associated with pressure on the genetics of the virus.

Although it was not possible to detect a correlation between the number of mutations and disease progression, the association among high frequencies of CTL escape mutations, elevated levels of CD8^+^ T lymphocytes and the predominant Th1 cytokine profile observed in ECs/VCs suggests that the CTL response in these individuals exerts considerable selection pressure on the virus. This pressure may contribute to the accumulation of mutations that, consequently, lead to effective control of viremia during infection through decreased viral fitness [60]. Overall, HLA subtyping may provide an important tool for a detailed analysis of the selection pressure exerted by the immune systems of these individuals.

The CTL-mediated immune response, which exerts selective pressure and induces escape mutations in HIV, is characterized by a strong and rapid response that occurs predominantly during acute/early infection [92, 93]. Thus, variability in the number and types of mutations appears to depend on the intensity of the individual’s initial immune response to HIV infection.

## Conclusion

Analysis of natural HIV-1 viremia controllers provides clues of the different pathways by which pathogenesis occurs, and one of these factors may be the pressure imposed by immune escape mutations. The present results show that individuals infected with HIV-1 subtypes B and F in the northern area of Brazil carry common and newly described mutations. Although the frequency did not differ between viremia controllers and noncontrollers, the findings reinforce the continuous need to unravel the complex disease development by the virus and the biological characteristics of the infected host.

## Data Availability

The datasets generated and/or analysed during the current study are available in the GenBank repository (Accession numbers: Gag: MT738717; MT738718; MT738719; MT738720; MT738721; MT738722; MT738723; MT738724; MT738725; MT738726; MT738727; MT738728; MT738729; MT738730; MT738731; MT749440. Nef: MT738732; MT738733; MT738734; MT738735; MT738736; MT738737; MT738738; MT738739; MT738740; MT758205. Rev: MT741971; MT741972; MT741973; MT741974; MT741975; MT741976; MT741977; MT741978; MT741979; MT741980. Tat: MT741981; MT741982; MT741983; MT741984; MT741985; MT741986; MT741987; MT741988; MT741989. Vif: MT749412; MT749413; MT749414; MT749415; MT749416; MT749417; MT749418; MT749419; MT749420. Vpr: MT749421; MT749422; MT749423; MT749424; MT749425; MT749426; MT749427; MT749428; MT749429; MT749430; MT758206. Vpu: MT749431; MT749432; MT749433; MT749434; MT749435; MT749436; MT749437; MT749438; MT749439; MT758207; MT758208; MT758209; MT758210; MT758211; MT758212).

## Abbreviations

HIV: Human immunodeficiency virus
CD4^+^TC: CD4^+^ T cells
AIDS: Acquired immune deficiency syndrome
LTNP: Long-term nonprogressors
HLA: Human leukocyte antigen
CTLs: Cytotoxic CD8^+^ T lymphocytes
EC: Elite controllers
VC: Viremia controllers
NVC: Nonviremia controllers
Th1: T helper 1
Th2: T helper 2
